# Effect of Multiyear Biodegradable Plastic Mulch on Soil Microbial Community, Assembly, and Functioning

**DOI:** 10.3390/microorganisms13020259

**Published:** 2025-01-24

**Authors:** Xiaowei Liu, Zongyu Wen, Wei Zhou, Wentao Dong, Huiqing Ren, Gang Liang, Wenwen Gong

**Affiliations:** 1 School of Biology, Food and Environment, Hefei University, Hefei 230601, China; liuxw@hfuu.edu.cn (X.L.); wenzongyu1998@163.com (Z.W.); 2 Institute of Quality Standard and Testing Technology, BAAFS (Beijing Academy of Agriculture and Forestry Sciences), Beijing 100097, China; 13501337719@163.com (W.D.); ren2020326@163.com (H.R.); 3 College of Sericulture, Textile and Biomass Sciences, Southwest University, Chongqing 400715, China; zowie2016@swu.edu.cn

**Keywords:** biodegradable plastics, soil microorganisms, microbial diversity, ecological functions, nutrient cycling

## Abstract

The increasing use of biodegradable plastic mulch like polybutylene adipate terephthalate (PBAT) has raised concerns about its long-term environmental impact. In this study, we investigated the effects of multiyear PBAT mulch application on bacterial and fungal communities, assembly mechanisms, and key ecological functions. The microbial community diversity and composition were significantly altered after multiyear biodegradable plastic mulching. We observed that PBAT treatment enriched specific bacterial genera, such as *Pantoea*, potentially involved in plastic degradation, and fungal genera like *Cephaliophora* and *Stephanosporaceae*, which may play a role in organic matter decomposition. A null model analysis revealed that bacterial community assembly was largely shaped by deterministic processes, with stronger environmental selection pressures in PBAT-treated soils, while fungal communities were more influenced by stochastic processes. In addition, multiyear PBAT mulch application also impacted the functionality of the soil microbial communities. PBAT exposure enhanced biofilm formation in aerobic bacteria, promoting aerobic degradation processes while also reducing the abundance of stress-tolerant bacteria. Additionally, PBAT altered key microbial functions related to carbon, nitrogen, and sulfur cycling. Notably, the fungal communities exhibited functional shifts, with an increase in saprotrophic fungi being beneficial for nutrient cycling, alongside a potential rise in plant pathogenic fungi. These findings underscore the multiyear ecological impacts of biodegradable plastics, suggesting microbial adaptation to plastic degradation and changes in key ecological functions, with implications for agricultural sustainability and bioremediation strategies.

## 1. Introduction

Plastic mulching is a widely used agricultural practice that helps conserve soil moisture, suppress weeds, and enhance crop yields [1,2]. However, the extensive use of conventional polyethylene (PE) mulch has raised significant environmental concerns due to its persistence in soil, leading to plastic pollution, particularly small plastic particles known as microplastics (MPs, <5 mm), which degrade soil health and ecosystem functions [3,4,5]. As a sustainable alternative, biodegradable plastic mulches (BDMs) have been introduced, which can decompose through microbial activity, offering the potential to mitigate the accumulation of plastic residues in agricultural soils [6,7,8,9].

Soil microbial communities play a central role in maintaining agricultural ecosystem health. They drive nutrient cycling, energy flow, and the structural integrity of soil, making them critical for soil fertility and productivity [10]. The diversity of these communities serves as a sensitive indicator of soil nutrient dynamics, reflecting subtle environmental changes and offering critical insights into soil functioning. Agronomic practices, such as plastic mulching, are known to influence soil microbial communities and dynamics [11,12]. For example, some studies have shown that conventional PE mulching can reduce microbial diversity by creating anaerobic microenvironments in the soil [13,14]. Additionally, MPs derived from mulch residues can act as unique microbial habitats, providing surfaces for biofilm formation and supporting the proliferation of specific bacterial taxa [15,16]. These findings highlight the complex interactions between plastic residues and soil microbial communities, emphasizing the need for a better understanding of how alternative mulching practices, such as the use of BDMs, influence soil microbiota over time.

Unlike conventional PE mulches, BDMs are designed to be tilled into the soil after use, where they are degraded under microbial activity, serving as a carbon and energy source for soil microbes [17]. This key difference has resulted in distinct impacts on soil microbial communities compared to PE mulches [18,19,20]. Additionally, soil bacterial and fungal communities exhibited different dynamics and ecological roles [21]. Investigating both groups simultaneously was therefore essential to comprehensively understand the ecological processes in soil systems [22]. While recent studies have explored the effects of BDMs on soil microbial diversity and functioning, most of these have focused on short-term impacts in controlled or single-year field studies [10,16,23,24]. The cumulative and dynamic impacts of multiyear BDM application on microbial community assembly and soil functioning remain largely unexplored, leaving critical knowledge gaps in the long-term sustainability of BDMs in agricultural systems.

This study investigated the effects of multiyear BDM application (1–3 years) on the dynamic change process of soil microbial communities through actual farmland soil sampling. Specifically, we aimed to (i) assess the impacts of varying durations of BDM use on the diversity, composition, and structure of soil bacterial and fungal communities and (ii) reveal how BDM application influences the microbial assembly processes and ecological functions. By addressing these objectives, this work provides novel insights into the ecological implications of BDM use and offers a foundation for guiding sustainable agricultural practices.

## 2. Materials and Methods

### 2.1. Samples and Reagents

Soil samples were gathered in July 2022 from a vegetable farm in Tongzhou District, Beijing (E 116°48′56.10″, N 39°52′04.74″), a pilot site for promoting BDM use in the region. Within the farm, plots covered with PBAT films for 1, 2, and 3 years were selected, and surface soil (0–10 cm) was sampled using stainless steel shovels. These samples were labeled as TZ1, TZ2, and TZ3, respectively. The PBAT films used in this study were sourced directly from local farmers and reflect the commonly used commercial biodegradable plastic mulches in the region. These films were not specifically standardized for this study, and detailed information regarding their exact composition and thickness was not available. While this introduced some variability, it also enhanced the real-world relevance of the findings as it aligned with typical agricultural practices. Adjacent plots without prior PBAT film application served as controls (CK). The soil samples were sieved through a 2 mm stainless steel mesh to remove stones and roots and then stored at −80 °C for further analysis.

### 2.2. DNA Extraction, PCR Application, and Sequence Analysis

DNA was extracted from soil samples using the E.Z.N.A.^®^ Soil DNA Kit (Omega Bio-tek, Norcross, GA, USA) following the manufacturer’s instructions. DNA quality and concentration were assessed via 1.0% agarose gel electrophoresis and a NanoDrop 2000 spectrophotometer (Thermo Scientific, Waltham, MA, USA). The extracted DNA was stored at −80 °C until further use.

The V3-V4 region of the bacterial 16S rRNA gene was amplified using primers 338F (5′-ACTCCTACGGGAGGCAGCAG-3′) and 806R (5′-GGACTACHVGGGTWTCTAAT-3′), while fungal ITS1 regions were amplified using primers ITS5 (5′-GGAAGTAAAAGTCGTAACAAGG-3′) and ITS2 (5′-GCTGCGTTCTTCATCGATGC-3′). PCR reactions were performed in a 20 μL volume containing Fast Pfu buffer, dNTPs, primers, polymerase, template DNA, and ddH_2_O. Cycling conditions included initial denaturation at 95 °C for 3 min, followed by 27 cycles of 95 °C for 30 s, 55 °C for 30 s, 72 °C for 45 s, and a final extension at 72 °C for 10 min. Amplified products were verified using 2% agarose gel electrophoresis, purified with a PCR Clean-Up Kit (YuHua, Shanghai, China), and quantified with a Qubit 4.0 Fluorometer (Thermo Fisher Scientific, USA). Sequencing libraries were prepared by pooling equimolar concentrations of purified PCR products and then sequenced on an Illumina NovaSeq platform by Magigene Biotechnology Co., Ltd. (Beijing, China).

### 2.3. Data Analysis

Raw sequencing data were processed on the Majorbio Cloud platform. Quality control involved demultiplexing, trimming primers, filtering low-quality reads, merging paired-end reads, and removing chimeras using fastp (v0.19.6) and FLASH (v1.2.7). Reads were retained if they were longer than 50 bp, had an average quality score ≥ 20, and allowed for up to two mismatches in primer regions. Sequences were clustered into operational taxonomic units (OTUs) at a 97% similarity threshold using UPARSE (v11), with the most abundant sequence being selected as the representative for each OTU. Taxonomy assignment was performed using the RDP Classifier (v2.2) against the SILVA (Release 138.1) and UNITE (Release 9.0) databases with a confidence threshold of 0.7.

Chloroplast sequences were manually removed, and diversity metrics were calculated using Mothur (v1.30.1). Alpha-diversity metrics (Chao1, Sobs, Shannon, Simpson, and Good’s coverage) and beta diversity metrics (Bray–Curtis dissimilarity) were estimated using the diversity plugin by rarefying each sample to 40,546 sequences. Alpha diversity index was calculated using the ASV table in QIIME2 and visualized as box plots. The taxonomy compositions and abundances were visualized using MEGAN [25] and GraPhlAn [26]. To investigate microbial community assembly, we used the beta Nearest Taxon Index (βNTI) and Raup-Crick (RCbray) based on the null model [27]. Values of |βNTI| ≥ 2 indicated deterministic processes, while |βNTI| < 2 suggested stochastic processes. We further categorized these processes into five types: homogeneous selection (βNTI ≤ −2), variable selection (βNTI ≥ +2), dispersal limitation (|βNTI| < 2, RCbray > 0.95), homogeneous dispersal (|βNTI| < 2, RCbray < −0.95), and undominated processes (|βNTI| < 2, |RCbray| ≤ 0.95). In addition, the phenotypic and functional predictions of bacteria were predicted using the Bugbase (https://bugbase.cs.umn.edu/index.html) and FAPROTAX (Version 1.2.4) databases, respectively [28,29]. FUNGuild database was used to classify the fungi in this study by ecological guild [30]. Statistical analyses were performed by using GraphPad Prism 10.1.

## 3. Results

### 3.1. Microbial Diversity

**Figure 1:**
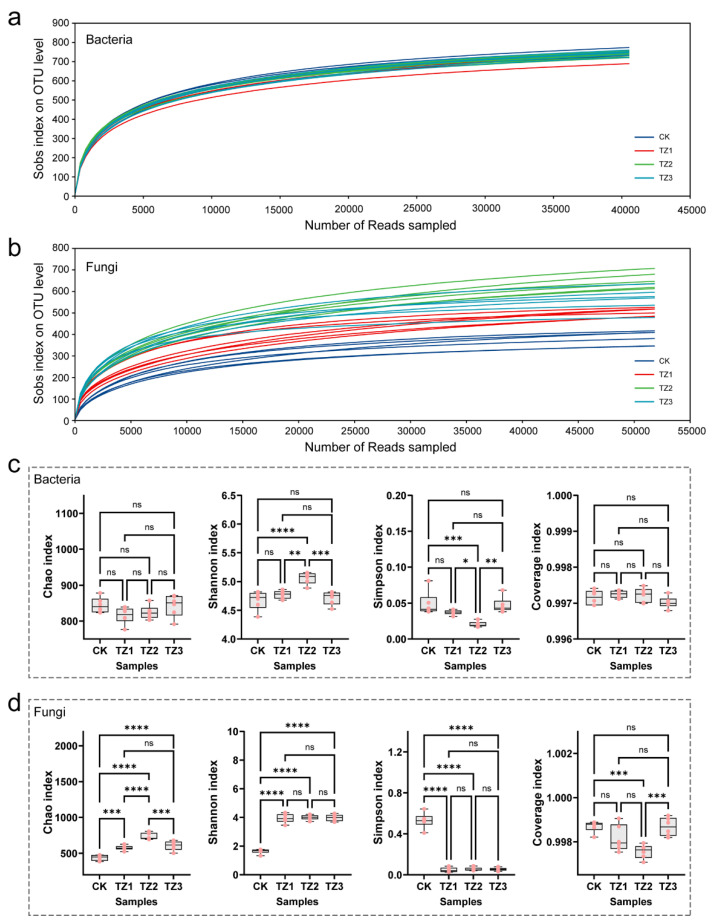
Rarefaction curves of bacterial (**a**) and fungal (**b**) communities based on observed OTUs of individual soil samples; boxplots of community α-diversity of bacteria (**c**) and fungi (**d**) (ns > 0.05, * *p* < 0.05, ** *p* < 0.01, *** *p* < 0.005, and **** *p* < 0.001).

Quality sequences recovered a total of 6805 bacterial and 2750 fungal OTUs, with rarefaction curves confirming that sequencing depth adequately captured the microbial community diversity (Figure 1a,b). We investigated the effects of 1-year (TZ1), 2-year (TZ2), and 3-year (TZ3) usage of biodegradable mulch on the soil microbial α-diversity. For bacterial communities, the Chao index, Sobs index, and Coverage index showed no significant differences between the control group (CK) and the treatment groups (Figure 1c). However, the Shannon index and Simpson index exhibited significant changes. Notably, no significant differences were observed between CK and TZ1. As the usage duration increased, the Shannon index of TZ2 significantly increased compared to CK (*p* < 0.001), while the Simpson index significantly decreased (*p* < 0.001). Interestingly, when the usage extended to 3 years (TZ3), these two indices returned to a non-significant difference relative to CK. In contrast, the fungal α-diversity was more significantly affected by the usage of biodegradable mulch (Figure 1d). Except for the Coverage index, all other indices—the Shannon index, Chao index, and Sobs index—showed significant increases in the TZ groups compared to the CK (*p* < 0.001), while the Simpson index significantly decreased (*p* < 0.001). In general, the bacterial and fungal communities displayed distinct response patterns to mulch usage. Bacterial communities showed a short-term perturbation followed by long-term recovery, while fungal communities exhibited sustained sensitivity. The results of the present study were consistent with the findings of other studies that the effects of BDMs on the soil microbial community diversity were significantly taxon- and time-dependent [16,23,31].

### 3.2. Microbial Community Composition

**Figure 2:**
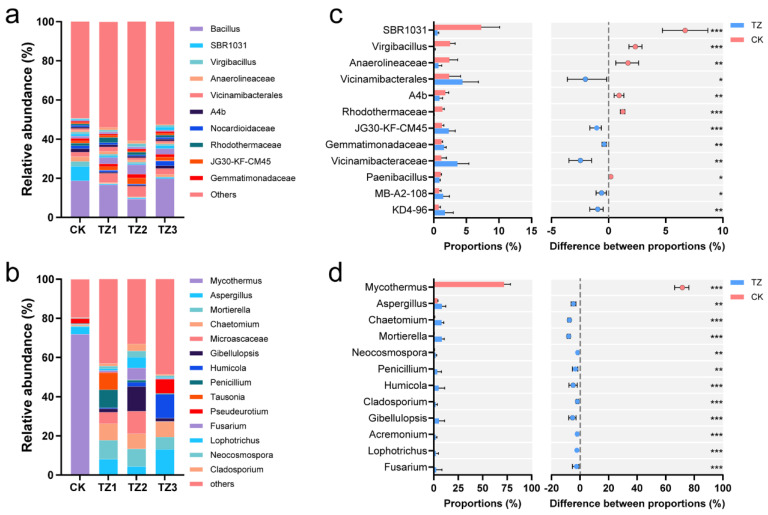
Genus-level species composition of bacteria (**a**) and fungi (**b**) in each soil sample, and effect of biodegradable mulching on species difference analysis of soil bacteria (**c**) and fungi (**d**) communities on genus level. Differences between groups were calculated using Wilcoxon rank-sum test. * *p* < 0.05, ** *p* < 0.01, and *** *p* < 0.001.

The compositions of bacterial and fungal communities at the genus level are shown in Figure 2. In the CK group, Bacillus was the most abundant bacterial genus, accounting for 18.8% of the total bacterial population. Although its abundance slightly decreased after PBAT treatment, Bacillus still remained the dominant genus (Figure 2a). It has been suggested that the diversity and abundance of carbon sources were the key factors in shaping microbial communities [32]. PBAT promoted the enrichment of some bacterial genera, including *Vicinamibacterales*, *Vicinamibacteraceae*, etc. (Figure 2c), suggesting that these bacteria are better equipped to utilize the carbon substrates associated with PBAT degradation or that their growth is favored by the modified soil environment. On the other hand, PBAT treatment led to a reduction in the abundance of certain bacteria, such as *SBR1031*, *Virgibacillus*, and *Anaerolineaceae*, which may be less adapted to the altered soil conditions induced by plastic residues [33].

For fungi, the changes at the genus level were even more pronounced (Figure 2b). As previous studies have shown, the addition of MPs may exert selective pressure on the soil microbiota, leading to changes in community structure over time [31,34]. The dominant fungal genus, *Mycothermus* (71.8%), significantly decreased in the PBAT-treated groups, with a dynamic shift to *Mortierella*, *Gibellulopsis*, and Aspergillus in treatments TZ1 to TZ3, indicating a shifting fungal community composition over time with PBAT application. However, the presence of PBAT inhibited the relative abundance of *Mycothermus*, potentially due to the inhibitory effects of plastic residues on its growth or metabolic activity. As a result, the relative abundance of other fungal genera increased (Figure 2d). Notably, genera such as Aspergillus, Penicillium [35], and Fusarium [36], which have been reported to possess plastic-degrading capabilities, showed a marked increase. This suggests that the introduction of PBAT may create a selective pressure that favors fungi capable of degrading plastic compounds, and PBAT may be utilized as a carbon source [37,38].

**Figure 3:**
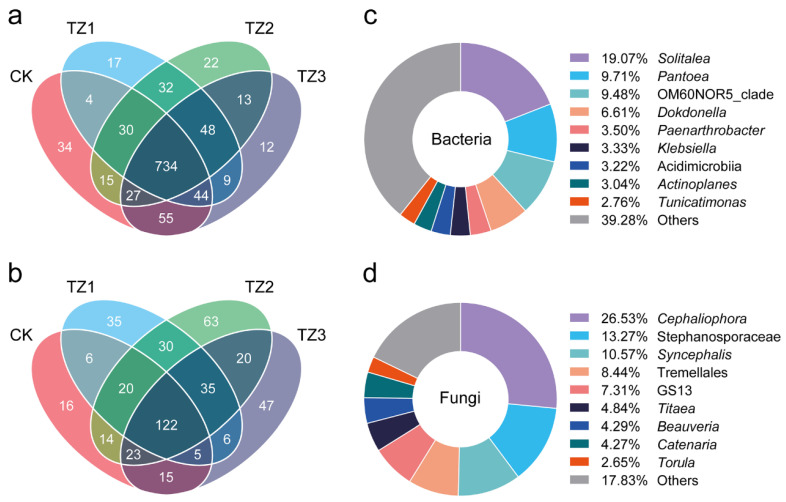
Venn diagrams showing the unique and shared bacterial (**a**) and fungal (**b**) OTUs in different groups; the composition of the shared 48 bacterial species (**c**) and 35 fungal species (**d**) in the TZ groups.

The Venn diagrams presented in Figure 3 illustrate the unique and shared bacterial (Figure 3a) and fungal (Figure 3b) OTUs across the different groups. The shared OTUs in all groups accounted for the biggest proportions, 734 and 122 for bacteria and fungi, respectively. Notably, it was found that 48 bacterial OTUs and 35 fungal OTUs are uniquely present in the TZ groups, indicating a shift in microbial community composition due to PBAT exposure. The presence of these unique OTUs in the TZ groups further emphasize the selective impact of PBAT on bacterial and fungal communities. These unique OTUs may represent taxa with metabolic capabilities to PBAT or other plastic-related compounds as carbon sources, or they may be adapted to thrive in environments where PBAT residues are present. For example, the unique bacterial genera *Solitalea* (Bacteroidetes phylum) and *Pantoea* (Proteobacteria phylum) were associated with organic compound degradation, and *Pantoea* species, in particular, have been shown to degrade plastics such as polyethylene [39,40]. Similarly, the fungal genera *Cephaliophora* (Ascomycota phylum) and *Stephanosporaceae* (Basidiomycota phylum), known for their roles in decomposing organic matter [41,42], may also contribute to the breakdown of plastic residues in the TZ groups. This observation was consistent with previous research that highlighted the enrichment of plastic-degrading or plastic-adapted microbes in soils contaminated with synthetic polymers [10,43]. Together, the unique bacterial and fungal OTUs in the TZ group underscore the microbial community’s potential to adapt to plastic pollution, both through selective enrichment and the establishment of specialized microbial taxa. These shifts in microbial diversity may have implications for soil health and microbial interactions, suggesting that PBAT not only alters the microbial composition but may also create niches for plastic-degrading microbes [44].

### 3.3. Assembly of Microbial Community

**Figure 4:**
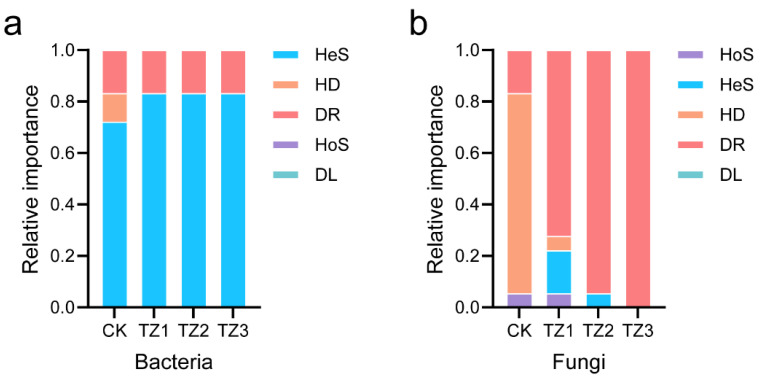
The fraction of assembly mechanism of soil bacterial (**a**) and fungal (**b**) communities in different treated soils based on the β-Nearest Taxon Index (βNTI). HeS, heterogeneous selection; HoS, homogeneous selection; HD, homogenizing dispersal; DL, dispersal limitation; DR, drift and others.

Microbial community assembly is shaped by both deterministic and stochastic ecological processes, though the degree of their influence can differ depending on the ecosystem [27]. Here, a null model analysis was conducted to explore the driving mechanisms of microbial community assembly. As shown in Figure 4a, heterogeneous selection (a deterministic process) dominated the bacterial community assembly in the CK group, contributing 72.22% of the total variation, and this was followed by drift and undominated processes (16.67%) and homogenizing dispersal (11.11%). In contrast, in PBAT-treated soils, the contribution of heterogeneous selection further increased to 83.33%, indicating a stronger deterministic influence. Therefore, the deterministic process played a more important part in influencing bacterial communities in PBAT-treated soils, in concordance with previous studies [45,46]. Figure 4b further reveals that fungal community assembly was more strongly influenced by PBAT, consistent with the result of alpha diversity and community structure (Figure 1 and Figure 2). In the CK group, fungal community was mainly shaped by homogenizing dispersal (a stochastic processes), whereas in the PBAT-treated soils, drift and other undominated processes played a dominant role over time. The stochastic processes had a stronger influence on fungal communities than on bacterial communities, suggesting that fungal communities were less affected by environmental factors, and random factors were the main influencing factors [46]. This suggests that bacterial communities are more influenced by environmental selection pressures, likely due to their more specific ecological functions, including PBAT degradation [47]. In contrast, fungal communities appear to be less constrained by environmental factors, demonstrating greater flexibility in response to fluctuating conditions, potentially due to their ability to exploit multiple nutrient sources [48]. These findings highlight the differential responses of bacterial and fungal communities to PBAT, with bacteria exhibiting a more deterministic assembly influenced by selective forces, while fungi are more susceptible to random processes.

### 3.4. Potential Microbial Functional Profiling Response to PBAT Films

**Figure 5:**
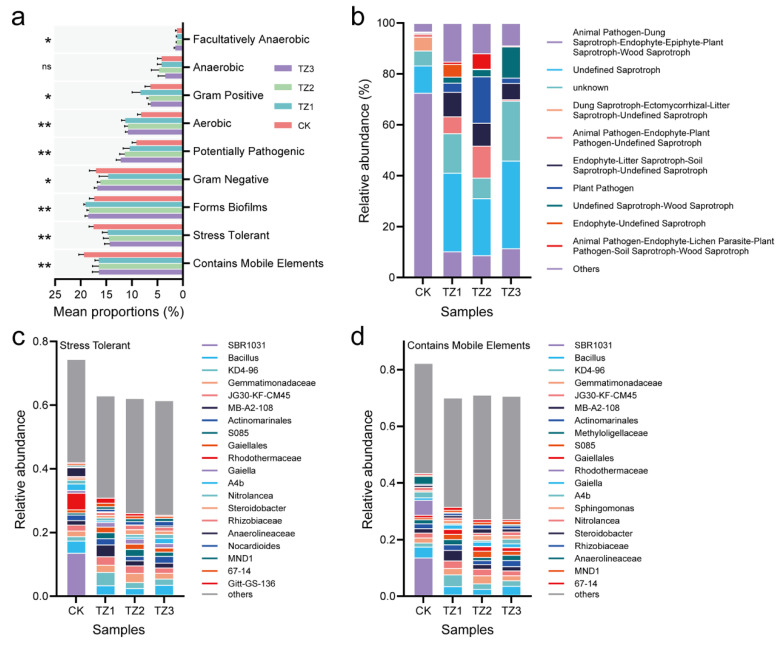
Phenotypic differences in colonies based on Bugbase prediction (ns > 0.05, * *p* < 0.05 and ** *p* < 0.01) (**a**) and prediction of fungal functions in different treated soils based on FUNGuild (**b**). Predicted relative abundance of bacteria in stress-tolerant (**c**) and bacteria containing mobile elements (**d**) based on BugBase database. For panel (**b**): AP, Animal Pathogen; DS, Dung Saprotroph; Ec, Ectomycorrhizal; En, Endophyte; Ep, Epiphyte; FP, Fungal Parasite; LP, Lichen Parasite; LS, Litter Saprotroph; PP, Plant Pathogen; PS, Plant Saprotroph; SS, Soil Saprotroph; US, Undefined Saprotroph; WS, Wood Saprotroph.

Based on predictions from the Bugbase database, long-term PBAT usage in soil notably impacted phenotypes of soil bacterial communities (Figure 5a). Specifically, the abundance of the aerobic and forms biofilms’ taxa was significantly higher in the long-term PBAT-treated soils than in the CK (*p* < 0.001), suggesting that PBAT residues may act as inducers for biofilm formation, consequently promoting the enrichment of aerobic bacteria. This enrichment could potentially enhance the efficiency of aerobic degradation processes as biofilms provide a conducive environment for microbial activity and metabolic processes associated with PBAT degradation [17]. In contrast, the abundance of stress-tolerant bacteria and those with mobile elements was significantly lower in soils with long-term PBAT usage (*p* < 0.01). This can be attributed to the dramatic reduction in certain species in long-term PBAT exposure, such as *SBR1031*, *Rhodothermaceae*, and *Anaerolineaceae* (Figure 5c,d), suggesting a change in the microbial community that may affect genetic exchange and adaptability within the soil ecosystem.

**Figure 6:**
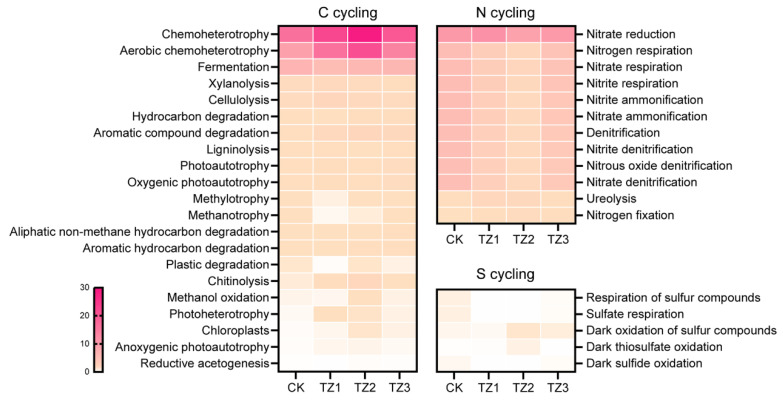
Nutrient cycling-related functions of bacterial communities as predicted by FAPROTAX.

In addition, FAPROTAX function prediction revealed that PBAT significantly influenced the nutrient cycling-related functions of soil bacterial communities (Figure 6). For C metabolism, there was a notable increase in the abundance of aerobic_chemoheterotrophy and chemoheterotrophy in the PBAT-treated groups, suggesting an enhancement in carbon metabolism. It is postulated that PBAT films may provide additional carbon sources, such as polymer residues or organic matter, thereby supporting bacterial growth and metabolic activities [49,50]. Significant increases were also observed in the abundance of aromatic_compound_degradation and aromatic_hydrocarbon_degradation, likely due to the aromatic components of PBAT, derived either from raw materials or degradation byproducts [38,51]. Conversely, several key functions related to N metabolism, including respiration, denitrification, and ammonification processes, were significantly reduced in the PBAT-treated groups. This reduction might indicate a limitation in nitrogen transformation and cycling, potentially caused by changes in the microbial community structure, changes in soil physical attributes, or the introduction of new carbon sources by the PBAT films [52,53]. Earlier research has documented comparable impacts of MPs on the C and N cycling in soil ecosystems [19,54]. In addition, predicted functional profiles related to sulfur cycling, such as sulfur compound oxidation, sulfate reduction, and sulfur respiration, were significantly influenced by PBAT. Other studies have also reported the impact of MPs on sulfur cycling, highlighting their potential to disrupt sulfur-related microbial processes in soil ecosystems [55,56,57].

Furthermore, the corresponding ecological functions of the fungi were obtained using FunGuild analysis (Figure 5b). Soil fungal communities after long-term PBAT application showed significant functional shifts. There was an increase in saprotrophic fungi (such as USs—Undefined Saprotrophs and WSs—Wood Saprotrophs) in the PBAT-treated groups compared to the CK. This increase suggested that PBAT film might alter the soil microenvironment, enhancing conditions for saprotrophic fungi growth by decomposing soil organic matter and releasing new nutrient resources [58]. This may be an unexpected benefit of the long-term use of PBAT film since saprotrophic fungi contribute to important ecosystem functions, such as C and N cycling, which are increasingly recognized to enhance soil health and agricultural sustainability [59,60]. In addition, functional categories related to animal pathogens (APs) and dung decomposition (DS—Dung Saprotrophs) exhibited decreased abundance in PBAT-treated soils, indicating possible inhibitory effects of PBAT on these fungi. Notably, an increase in plant pathogens (PPs) was observed, which highlighted the potential risk of promoting plant pathogenic fungi due to PBAT usage. This aligns with concerns raised in previous research, which indicated that MPs can lead to an increased proportion of bacterial pathogens in soil or serve as carriers of bacterial pathogens [57,61]. This study suggested that a similar risk might apply to fungal pathogens. Recent research by Qi et al. [62] found that biodegradable plastics lead to the significant enrichment of two fungal genera (*Rhizoctonia* and *Fusarium*), opportunistic plant pathogens known for causing significant crop diseases (such as root rot, stem rot, and seed decay). Therefore, further investigation is required to understand how PBAT facilitates the proliferation of plant pathogens and the implications for agricultural ecosystems, considering the potential ecological risks alongside the benefits.

## 4. Conclusions

While biodegradable plastics, such as PBAT, are promoted as eco-friendly alternatives to conventional plastics, concerns about their environmental impact persist. Based on a field sample analysis, this study revealed the significant effects of PBAT biodegradable film on soil microbial communities and ecosystem functions. We found that PBAT exposure significantly altered both bacterial and fungal communities, with specific genera becoming more abundant in PBAT-treated soils. Bacterial community assembly was primarily driven by deterministic processes in bacterial communities, with stronger environmental selection pressures in the PBAT-treated soils compared to the CK group. In contrast, fungal communities were more influenced by stochastic processes, reflecting their greater flexibility in response to fluctuating environmental conditions. The shifts in community composition (key species) were accompanied by changes in microbial ecological functions, particularly in nutrient cycling. Notably, PBAT exposure enhanced carbon metabolism and aromatic compound degradation but inhibited nitrogen transformations and disrupted sulfur cycling.

Our findings underscore the complex, lasting ecological effects of multiyear PBAT exposure on soil ecosystems. Future research should focus on exploring the molecular and biochemical pathways involved in plastic degradation by soil microbes, as well as investigating the potential risks associated with the accumulation of plastic residues in soils. Additionally, the interaction between microbial communities and the plant rhizosphere in the presence of biodegradable plastics warrants further investigation to assess potential impacts on crop health and soil fertility. Overall, these results highlight the need for a comprehensive evaluation of the long-term impacts of biodegradable plastics on soil health and agricultural sustainability.

## Data Availability

The original contributions presented in this study are included in the article. Further inquiries can be directed to the corresponding authors.
